# Practical use of apomorphine infusion in Parkinson’s disease: lessons from the TOLEDO study and clinical experience

**DOI:** 10.1007/s00702-023-02686-7

**Published:** 2023-09-01

**Authors:** Tove Henriksen, Regina Katzenschlager, Roongroj Bhidayasiri, Harry Staines, Donna Lockhart, Andrew Lees

**Affiliations:** 1 Department of Neurology, Movement Disorder Clinic, University Hospital of Bispebjerg, 2400 Copenhagen, Denmark; 2 Department of Neurology and Karl Landsteiner Institute for Neuroimmunological and Neurodegenerative Disorders, Klinik Donaustadt, Vienna, Austria; 3 Chulalongkorn Centre of Excellence for Parkinson’s Disease & Related Disorders, Department of Medicine, Faculty of Medicine, Chulalongkorn University and King Chulalongkorn Memorial Hospital, Thai Red Cross Society, Bangkok, Thailand; 4 The Academy of Science, The Royal Society of Thailand, Bangkok, Thailand; 5 Sigma Statistical Services, Balmullo, UK; 6 Britannia Pharmaceuticals Limited, Reading, Berkshire UK; 7 University College London Institute of Neurology and the National Hospital, Queen Square, London, UK

**Keywords:** Parkinson’s disease, Apomorphine infusion, Clinical practice

## Abstract

Subcutaneous apomorphine infusion is a device-aided therapy for Parkinson’s disease that can be considered when motor fluctuations become persistent and are no longer adequately controlled by oral/transdermal medication. Apomorphine infusion is less invasive than enteral levodopa, deep brain stimulation or focused ultrasound, and is often indicated even when neurosurgical approaches are contraindicated. This article aims to provide practical guidance for doctors and nurses initiating and treating patients with apomorphine infusion, and is based on both trial data and clinical experience from movement disorders specialists. A post hoc analysis of data from the TOLEDO randomized clinical trial of apomorphine infusion was conducted along with an analysis of ‘real world’ experience from 13 movement disorders specialists using a questionnaire that focused on starting patients on apomorphine infusion. Practical guidelines for starting treatment with apomorphine infusion are provided taking into consideration the regional disparities in healthcare. Apomorphine infusion is straightforward to administer but to be successful it requires concordance from the patient and family, and clinical support from an experienced team of doctors and nurses, particularly in the early months of treatment.

**Supplementary Information:**

The online version contains supplementary material available at 10.1007/s00702-023-02686-7.

## Introduction

When motor fluctuations develop in levodopa-treated patients with Parkinson’s disease (PD), adjuvant medications, when used judiciously, can reduce fluctuations and dyskinesias and improve quality of life and activities of daily living (Chaudhuri et al. 2016; Drapier et al. 2016; Houvenaghel et al. 2018; Meira et al. 2021). If disabling fluctuations persist despite optimized oral medication, device-aided therapies should be considered.

Subcutaneous apomorphine infusion has been used for over 30 years to maintain patients in the ON state while minimizing dyskinesia (Manson et al. 2002; Katzenschlager et al. 2005; Jenner and Katzenschlager 2016). It is the only dopamine analog with an equivalent efficacy to levodopa (Jenner and Katzenschlager 2016).

According to the UK’s National Institute for Health and Care Excellence (NICE) guidelines, apomorphine should be considered as part of ‘best medical therapy’ before options that involve surgical interventions, such as deep brain stimulation (DBS) and gastro-jejunostomy used to deliver levodopa infusion (National Institute for Health and Care Excellence 2017).

The efficacy and safety of apomorphine have been reported in many open-label and observational studies and confirmed in clinical practice (Jenner and Katzenschlager 2016; Garcia Ruiz et al. 2008; Sesar et al. 2017; Fox et al. 2018; van Laar et al. 2010). The TOLEDO study was the first randomized, placebo-controlled, double-blind, multicenter trial with apomorphine to provide Level 1 evidence of benefits in PD patients with persistent motor fluctuations despite optimized oral/transdermal medication (Katzenschlager et al. 2018, 2021), showing a clinically meaningful OFF-time reduction versus placebo, mirrored by an increase in ‘good quality’ ON time. The 52-week open-label phase showed that these effects were sustained (Katzenschlager et al. 2021).

Despite its proven efficacy and safety, apomorphine infusion is still underused and often used too late to be optimally effective (Auffret et al. 2018a, 2018b; Poewe and Wenning 2000). One explanation may be that it is perceived as complicated to initiate and supervise. Patients’ misperceptions of infusions as ‘last resort’ therapies may also play a role (Bhidayasiri et al. 2019).

We aimed to address these concerns and to provide practical guidance for apomorphine infusion use, drawing on published evidence, the procedures in the TOLEDO study, and clinical experience from movement disorders specialists around the world.

## Methods

Two approaches were taken:

1. A post hoc analysis of the TOLEDO study data of practices relating to initiation, dose optimization, and changes in concomitant medications.
2. An analysis of responses to a questionnaire from movement disorders specialists experienced in the use of apomorphine.

### Post hoc analyses of the TOLEDO study

The treatment practices were analyzed. Initiation during the double-blind phase followed strict protocol requirements (Katzenschlager et al. 2018); whereas during the open-label phase, patients were initiated according to local practice/guidelines (Katzenschlager et al. 2021). The following analyses were undertaken:

1. *Anti-emetic use:* The days of use of an anti-emetic were noted.
2. *Titration during the first 10 days*: The protocol included an inpatient stay, or day-case admission for at least 8 h, for up to 10 days. Data from the first 10 days of the double-blind and open-label phases were analyzed for average doses.
3. *Time to stable dose and stable dose achieved*: For the double-blind phase, concomitant medications could only be changed during the first 4 weeks. In the open-label phase, this was done based on local guidelines/practice. Consequently, a stable dose was defined as the longest period unchanged after the first 4 weeks. Time to stable dose was defined as the days from first administration to the first day of the maintenance dose.
4. *Concomitant anti-PD medication changes*: The number of patients whose concomitant anti-parkinsonian drugs were increased or decreased was determined. ‘When required’ medication doses were excluded. Increases and decreases were defined as changes in the levodopa equivalent daily dose (LEDD) of at least 10%. A stable dose was defined as < 10% increase or decrease. Time points were 6 weeks into the double-blind phase, the end of the double-blind and the end of the open-label phase.
5. *Hours of infusion*: The median number of infusion hours during the double-blind phase (when subjects were required to monitor daily use) was calculated.

### Questionnaire

A questionnaire was developed by a clinician (TH) and a statistician (HS) and sent to movement disorders specialists experienced in the use of apomorphine. These movement disorder specialists were chosen due to their extensive experience with apomorphine infusion therapy. They represent Thailand, Australia, UK, Spain, Portugal, France, Italy, Austria, Germany, Sweden, Norway, and Denmark, and are listed in full in the supplementary material. A total of 12 questionnaires were sent out. Since the primary author of this article (TH) also completed one, a total of 13 questionnaires were included in the analysis. One responder chose to pass on the questionnaire to a local colleague who was more experienced with apomorphine infusion. The response rate was 100%.

The questionnaire included the following aspects:

- Optional trial administration of apomorphine
- Anti-emetic use
- Initiation: inpatient or outpatient; starting dose; dose increments
- Reduction of concomitant medications
- Monitoring on established treatment
- Use of the pump’s bolus function

The full questionnaire can be found in the *Supplementary Material* (see S1).

## Results

Thirteen movement disorders specialists with experience of infusion treatments completed the questionnaire. Based on these and the post hoc TOLEDO analyses, recommendations are given for best clinical practice when starting patients on apomorphine and ongoing monitoring.

### General considerations when starting apomorphine

Table 1 summarizes the findings from the TOLEDO study and the survey.

1. As with other device-aided therapies, a detailed discussion needs to take place with the patient and their family/carer(s) regarding the pros and cons of apomorphine infusion to determine whether it would fit their personal circumstances.
2. In some countries, specially trained PD nurses (Bhidayasiri et al. 2016a), provided by the pharmaceutical company, are available to support the hospital team.
3. Funding for apomorphine infusion varies across healthcare systems; centers need to ensure that funding or reimbursement approval is in place.
4. It is advisable to establish local guidelines or standard operating procedures to ensure communication and coordinated action between patient, carers and healthcare professionals.
5. An initial trial of subcutaneous apomorphine injection before starting infusion treatment is sometimes undertaken to confirm dopaminergic responsiveness and enable patients to experience the effects of the medication. This may also provide an estimate of the hourly dose needed. If a test dose is performed, motor function is assessed in an OFF state and is then followed by gradually increasing doses of apomorphine, until a clear switch to an ON state occurs. For dose finding, the prescribing information recommends intervals of 30–40 min between each apomorphine dose increase. A more rapid titration scheme has been proposed to avoid prolonged OFF states (Hagell and Odin 2014) but has not yet been compared for safety with the recommended advice. In our survey, 9 of the 13 respondents (69%) stated that they routinely used a trial exposure (5 in an inpatient setting). The most common maximum dose was 2 mg or less, although some used up to 6 mg.

**Table 1:** Suggested stepwise initiation protocol for apomorphine infusion

1	Initiation setting	Can be undertaken in a hospital outpatient or day-case setting, usually over 5–10 days. Subsequent adaptations may take several weeks, especially if several oral drugs are reduced in a step-wise manner
2	Anti-emetic medication	Should be administered according to local guidelines, if available ECG monitoring, for the QTc interval, is recommended prior to and during the initiation of domperidone It is recommended to commence the anti-emetic domperidone at least 2 days prior to initiating apomorphine, at a dose of 10 mg three times/day Due to the risk of QTc prolongation, domperidone should be used at the lowest effective dose and for the shortest possible duration (see https://www.ema.europa.eu/en/medicines/human/referrals/domperidone-containing-medicines)
3	Establishing a stable apomorphine dose	Starting dose: a starting dose of up to 1 mg/hour on day 1, with daily rate increases of 0.5–1 mg/hour, until the patient is stabilized on an effective, tolerated dose with individually adjusted concomitant oral medication Each patient’s stable apomorphine dose will depend on individual efficacy and tolerability
4	Reducing or discontinuing concomitant anti-PD medications	Patients treated with apomorphine continuous infusion can substantially reduce their concomitant oral PD medication, and in some cases discontinue them. This is a specific treatment goal in patients with troublesome dyskinesias Once a patient is established on apomorphine, the suggested procedure is to reduce oral PD medications in the following order: Dopamine agonists Monoamine oxidase type B (MAOB) inhibitors Catechol-O-methyl transferase (COMT) inhibitors Oral levodopa dose, then frequency
5	Infusion hours	Usually during daytime, i.e., around 16 h/day. In patients with troublesome nocturnal OFF symptoms, 24-h use may helpful
6	Bolus dose function	Provides flexibility to give additional medication if needed but without increasing the overall dose per hour Frequent use of the bolus dose function suggests that the hourly flow rate may need to be increased

### Setting for apomorphine infusion initiation

*TOLEDO study*: Treatment was initiated during a 5–10-day inpatient hospital stay; in centers where outpatient initiation was already standard practice, repeated day-case admissions were permitted.

*Questionnaire responses*: In clinical practice, apomorphine infusion initiation occurs in both inpatient and outpatient settings, and in some cases with a mixture of the two even at the same center. Ten respondents admitted all patients for initiation and 2 combined in- and outpatient initiation.

*Recommendations*: Outpatient initiation is often straightforward to implement but there may be specific reasons why hospital admission is preferable. Although there is no direct comparison of settings, inpatient initiation may make the patient feel more confident, allow repeated training in handling the device, and give staff the opportunity to make early observations on tolerability and difficulties the patient may have in initiating the pump. Initiation of treatment can usually be completed in 5–10 days, after which less frequent visits are required for further adjustments.

### Anti-emetic use

Patients are often pre-treated with domperidone prior to apomorphine initiation to avoid nausea. Domperidone has been associated with a small risk of QTc interval prolongation (European Medicines Agency 2014) although study results are equivocal. A systematic review reported an increased risk of sudden cardiac death and ventricular arrhythmia with domperidone use compared with non-use (Ou et al. 2021). An earlier study in PD patients identified a significant risk only in those with a history of cardiovascular disease (Renoux et al. 2016). Recent findings from a single-center study of domperidone use (30–80 mg daily for up to one year) for gastroparesis in 246 patients observed QTc prolongation in 6%; in none was this found to be clinically significant (Field et al. 2019).

*TOLEDO study*: Anti-emetic pre-medication was administered according to local standards and the investigator’s judgement. The recommended dose of domperidone was 10 mg three times a day starting 3 days before the infusion. In the double-blind phase, 33 apomorphine-treated patients (61.1%) in the safety set received anti-emetic treatment; 19 patients did not take domperidone. Of the 33 who received domperidone, 10 stopped within 2 weeks and 17 continued for 12-weeks (51.5%). All patients in the open-label phase safety set (*n* = 84) were treated with apomorphine. Of these, 41 (48.8%) did not take domperidone. For those who did, the timing of discontinuation varied considerably; only 5 still used it at the end of the study.

*Questionnaire responses*: Anti-emetic medication was generally given between 1 and 3 days prior to apomorphine initiation (10/13 responses). Only one respondent reported not using anti-emetic medication at all. The most usual dose was 10 mg domperidone three times per day (10/13 responses), although two used 20 mg three times per day. Respondents adhered to the precaution of carrying out ECGs prior to and during the initiation, and used it at the lowest effective dose for the shortest possible duration (usually < 1 week), as per the European Medicine Agency (EMA) recommendations (European Medicines Agency 2014). The duration of use varied but it was usually stopped within 14 days (8/13 responses). Four respondents continued it for 21 days.

*Recommendations*: Clinicians should review the patient’s medical history, medications, and ECG. If there is no prior history of nausea, domperidone may not be needed at all. It is also contraindicated in severe hepatic impairment, in conditions where cardiac conduction is or could be impaired, and when co-administered with QTc-prolonging medications or potent CYP3A4 inhibitors (European Medicines Agency 2014). If needed (and available), domperidone should be used at the lowest effective dose for the shortest possible time, as per EMA recommendations. Our results suggest most patients only require it for a few days.

### Titration during the first 10 days

**Fig. 1:**
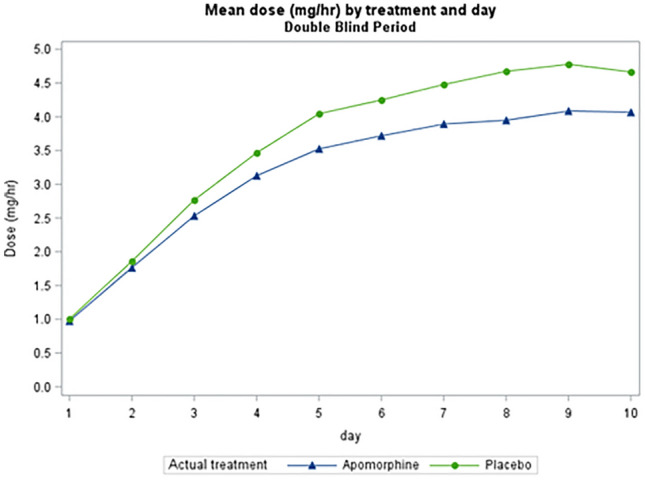
Titration regimen during the first 10 days of the TOLEDO study double-blind phase

**Fig. 2:**
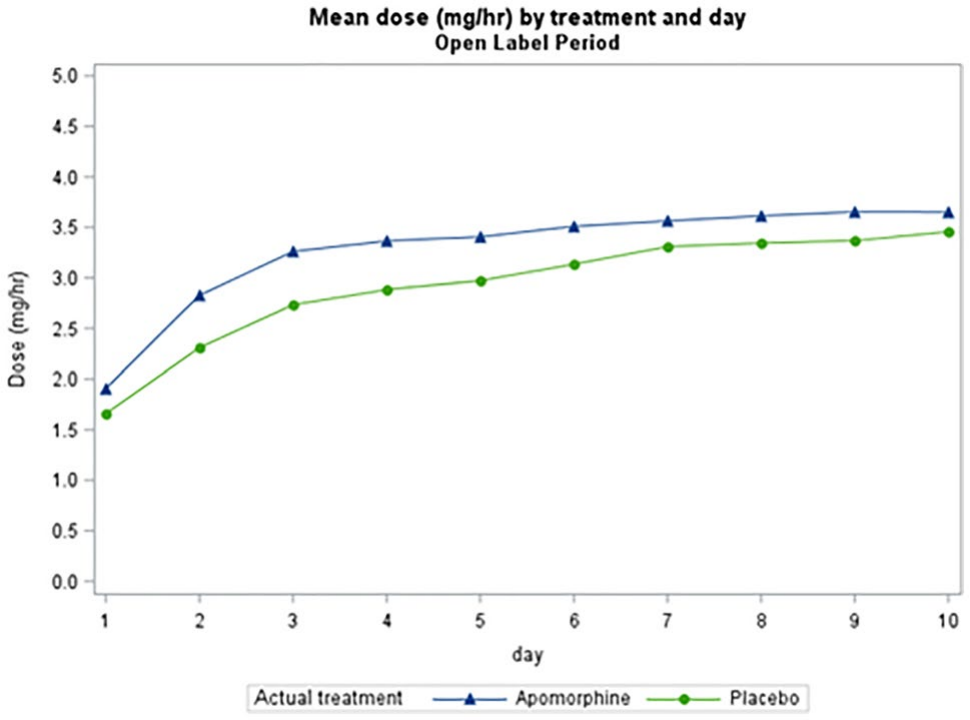
Titration regimen during the first 10 days of the TOLEDO study open-label phase. Note that during the open-label phase, while all patients were treated with apomorphine infusion, results are displayed according to the treatment they received during the double-blind phase

*TOLEDO study*: Figs. 1 and 2 show the mean hourly apomorphine/placebo dose during the first 10 days. Median starting dose in the double-blind phase for apomorphine-treated patients (*n* = 54) was 1 mg/hour, as specified in the study protocol, and median dose at Day 10 was 4.0 mg/hour (*n* = 53). In the open-label phase, median starting dose was 1.25 mg/hour (*n* = 84) and median dose on Day 10 was 3.50 mg/hour (*n* = 83).

*Questionnaire responses*: The starting dose was no more than 2 mg/hour (8/13 responses), although it varied considerably.

*Recommendations*: There are various titration options in clinical practice: a quick titration, most commonly as an inpatient, starting at 0.5–1.0 mg/hour, with increments of 0.5–1.0 mg every few hours or at daily intervals, depending on tolerability or increased dopaminergic effects such as dyskinesias; or a slower titration, most suited for outpatient initiation, with increments of 1.0 mg/hour at intervals depending on individual response and tolerability but are usually between several days and around 1 week. If no anti-emetic is available and in an outpatient setting, these intervals may need to be longer (1–2 weeks).

### Time to stable dose and stable dose achieved

**Fig. 3:**
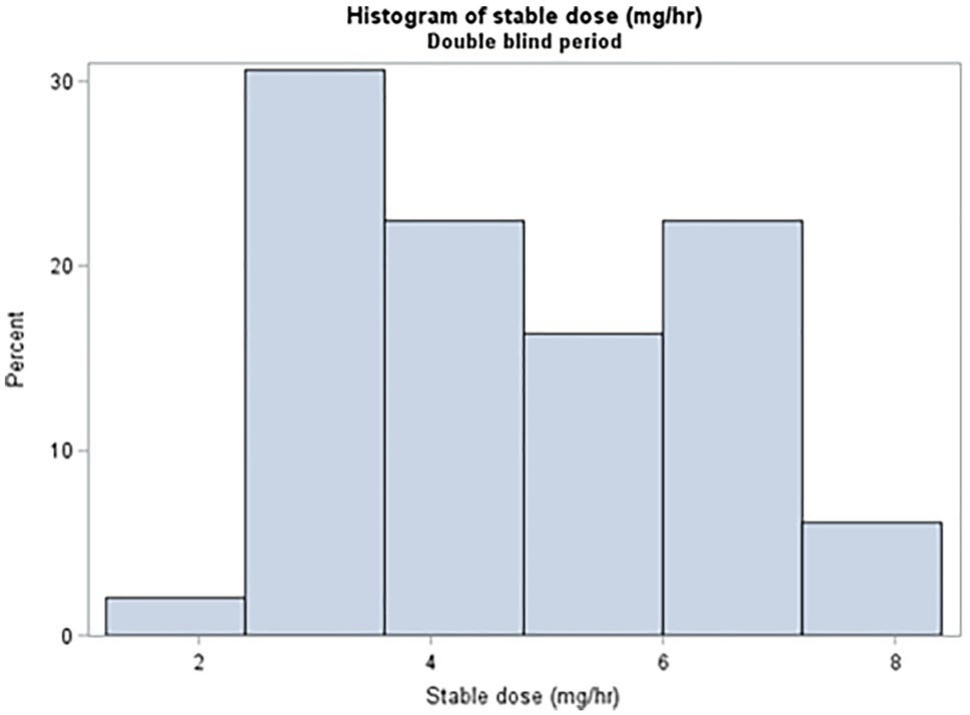
The stable dose of apomorphine achieved during the TOLEDO study double-blind phase

**Fig. 4:**
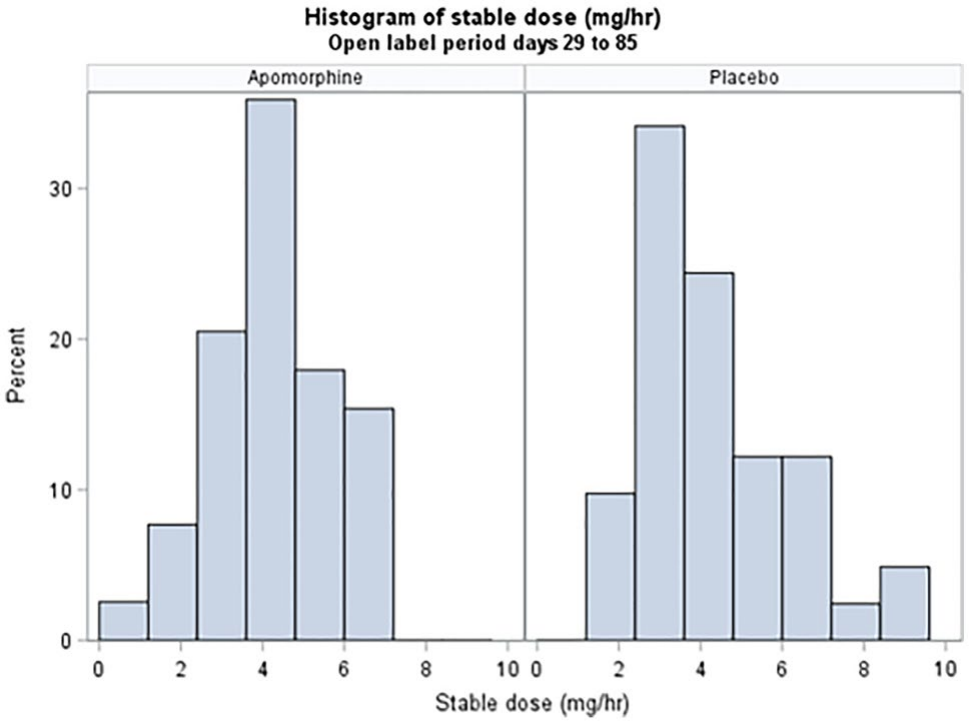
Stable dose of apomorphine achieved during the TOLEDO open-label phase. Note that during the open-label phase, while all patients were treated with apomorphine infusion, results are displayed according to the treatment they received during the double-blind phase

*TOLEDO study*: Figs. 3 and 4 show stable maintenance dose distribution. The median stable dose was 4.5 mg/hour and 4.35 mg/hour in the double- and open-label phases, respectively. Median time to achieve a stable dose was 29 days for APO-treated patients in the double-blind phase (*n* = 54). In patients who achieved a stable dose (*n* = 49), 40.8% took longer than 29 days. In the open-label phase (*n* = 80), median time to achieve a stable dose was 60 days.

*Questionnaire responses:* A stable apomorphine dose is generally achieved within 3 weeks (9/11 responses) although this can take longer (more than 8 weeks). Respondents stated that the time to stable dose was influenced by the speed of dose increases (‘fast’ or ‘slow’ approach) and also the speed of oral dopaminergic medication reduction. The stable maintenance flow rate is commonly between 3 and 7 mg/hour (10/11 responses). Males often need a slightly higher dose than females. Respondents commented that variable flow rates were often used, with lower rates at night.

*Recommendations*: Each patient’s stable apomorphine dose will depend on individual efficacy and tolerability, and on the patient’s wishes: Some are keen to achieve a substantial oral reduction, always a treatment goal in patients with dyskinesias. In contrast to the randomized study, in clinical practice, there is no time limit to dose adjustments, and this may take longer than the 4 weeks specified in the study protocol. The aim is an effective, well-tolerated dose with individually adjusted concomitant oral medication.

### Reductions and discontinuation of concomitant anti-PD medication

Early dopaminergic side effects are often wrongly attributed to the new therapy, whereas they may indicate that concomitant anti-PD medications should be reduced.

*TOLEDO study:* Dopaminergic effects (particularly dyskinesias, nausea, orthostatic hypotension, or sleepiness) were indicators for the clinician to consider reducing or discontinuing concomitant oral PD medications. This was done in the following prescribed order:

1. Dopamine agonists
2. Monoamine oxidase type B (MAOB) inhibitors
3. Catechol-O-methyl transferase (COMT) inhibitors
4. Levodopa dose, then dose frequency

*Questionnaire responses*: The vast majority (11/13 responses) recommended reducing oral dopamine agonists first to avoid dual dopamine agonist therapy for longer than necessary.

*Recommendations*: If dopaminergic side effects, such as increased dyskinesias, occur on apomorphine, then concurrent oral medication should be gradually reduced, usually in the order suggested in Table 1. This also has the advantage of reducing the number of oral medications and doses taken during the day. A sudden total withdrawal of oral medications is contraindicated, and in the case of dopamine agonists may precipitate dopamine agonist withdrawal syndrome if the apomorphine dose is not yet high enough to compensate (Yu and Fernandez 2017; Koschel et al. 2022). Dyskinesia severity determines the optimal degree of dose reduction. To treat OFF periods, the reduction in oral drugs may only need to be small; however, in a patient with troublesome dyskinesia, gradual withdrawal of all daytime oral medication may be required with the exception of a night-time and early-morning levodopa dose. If the patient continues to experience tolerability issues despite marked oral medication reductions, the apomorphine dose should then be slowly decreased. Conversely, if side effects are reduced following oral drug alterations, but the clinical effect of apomorphine infusion is still sub-optimal, apomorphine dosage should be slowly increased. In patients with troublesome nocturnal OFF symptoms, including sleep fragmentation, 24-h use can be helpful.

If apomorphine infusion is only used during daytime, nocturnal parkinsonian symptoms may be alleviated by using levodopa at bedtime; long-acting oral dopamine agonists or rotigotine are also alternatives. If dyskinesias are still troublesome while using apomorphine infusion, amantadine can be introduced or increased. During the early treatment phase, regular clinic visits are needed to achieve the best results and encourage the patient to take control over the administration of their treatment.

### Hours of infusion

*TOLEDO study*: The median daily hours of infusion for apomorphine-treated patients in the double-blind phase was 14.39.

*Questionnaire responses*: Median infusion duration was 12–16 h (7/13 responses), but some use longer durations (> 16 h; 4/13 responses). Some use apomorphine infusion for 24 h, for example for nocturnal disabling OFFs, muscle spasms, or painful dystonia.

*Recommendations*: The usual aim is for a waking day infusion of around 16 h, depending on each patient’s personal schedule and preference. Around-the-clock use may be helpful for troublesome nocturnal OFF symptoms. In addition, a recent multicenter, randomized, controlled, double-blind, crossover study (APOMORPHEE) reported that night-time only administration of apomorphine infusion at was able to improve sleep disturbances in patients with insomnia (De Cock et al. 2022).

### Monitoring of patients established on treatment and management of adverse events

Frequent monitoring after pump initiation is the key to successful long-term treatment. Patients should be made aware of possible adverse events and instructed to make contact with the doctor or nurse specialist if they have concerns. The most frequently reported adverse events are panniculitis (subcutaneous nodules, skin induration, erythema, and needle site tenderness), yawning, nausea, vomiting, sedation, somnolence, dizziness, unwanted penile erections, and neuropsychiatric disturbances (Katzenschlager et al. 2018; Borgemeester et al. 2016).

*TOLEDO study*: Long-term apomorphine infusion was well tolerated with adverse events and tolerability in line with previous observational studies. The most common treatment-related adverse events were mild or moderate infusion site reactions, nausea, and somnolence. While impulse control disorders (ICDs) are associated with all dopamine agonists, evidence suggests that the frequency is greater in those with preferential affinity to the dopamine D3 receptor and lower for apomorphine (Seeman 2015). Overall, the risk of developing ICDs is reported to be relatively low for infusion therapies, including apomorphine Todorova et al. 2015). Results from the TOLEDO study supported this, with observed ICD cases all being mild in severity, some reversible, and occurring uncommonly (less than 10% of patients) (Katzenschlager et al. 2018, 2021). There were no changes in the screening scale used for evaluating ICDs throughout the study.

*Questionnaire responses*: Continuing patient follow-up on stable maintenance dose (usually every 3–6 months) is considered best practice, including clinical evaluation and inspection of the skin. Many centers perform lying and standing blood pressure recordings and electrocardiography. Full blood counts with hemoglobin, reticulocytes, and Coombs antiglobulin test are performed every 3–12 months, but respondents recommended informing patients and carers about symptoms of adverse events, including the very small risk of hemolytic anemia.

*Recommendations*: One advantage of apomorphine infusion over other device-aided therapies, such as enteral levodopa infusion, is that it is easy to discontinue if the response is unsatisfactory or if intolerable adverse events occur. Skin nodules and irritation are rarely a cause for discontinuation (Bhidayasiri et al. 2016b). Massaging the insertion site after needle withdrawal, using finer or Teflon needles, antiseptic insertion techniques, therapeutic abdominal wall ultrasound, or, in selected cases, hydrocortisone injections can all reduce troublesome skin nodules.

Orthostatic hypotension occurs in ≤ 1% of patients but is usually mild and transient (Bhidayasiri et al. 2016b). Monitoring patients’ fluid intake and other non-pharmacological measures can be helpful. Hemolytic anemia, indicated by a falling hemoglobin level, a positive Coombs test and other laboratory changes (such as increased lactate dehydrogenase, decreased haptoglobin and increased reticulocytes), is a rare serious adverse effect. In clinical practice, in addition to blood checks, clinical signs of anemia should be looked for and patients should be educated about symptoms of anemia, such as tiredness and breathlessness and pallor, as hemolysis can occur quickly and may not be captured by occasional blood screening. Specialist hematological advice should be sought if hemolytic anemia is suspected and corticosteroid therapy or apomorphine withdrawal is required.

### Bolus function

The mini-pump has a bolus function, set by the clinician, which allows patients to administer additional rescue doses as needed for anticipated or prolonged OFF episodes.

*TOLEDO study*: Using the bolus function was allowed during the open-label phase. Bolus use varied considerably—some centers made it available for all patients, while some discouraged it (unpublished; Britannia Pharmaceuticals Limited data on file). The majority of patients in the open-label phase who had the bolus function set had this done in the first week of treatment, so it is unlikely that this explained any lack of efficacy. Post hoc analysis also showed that whether the bolus function was set or not did not depend on the treatment received during the double-blind phase or a range of demographic and baseline characteristics (unpublished; Britannia Pharmaceuticals Limited data on file).

*Questionnaire responses*: All respondents reported that at least some of their patients used the bolus function, most often to set up the pump in the morning, or for OFF episodes around mealtimes.

*Recommendation*: Occasional use of the bolus function of the apomorphine infusion pump is recommended, as it increases the efficacy of treatment, but patients need to be educated on situations where its use may be appropriate. Frequent bolus use (> 3 times daily) indicated the hourly flow rate should be increased.

## Discussion

We have based this report on unpublished data collected from the TOLEDO trial and ‘real-world’ experience with apomorphine collected in a semi-structured way from movement disorders specialists. Patients were highly selected in the randomized clinical trial and monitored more closely than in clinical practice. The specifications of the TOLEDO protocol limited individual clinical decision making, for example, centers were not permitted to continue dose adjustments after the first four weeks of double-blind treatment. These differences were reflected in the questionnaire responses. Nevertheless, in both settings a stable apomorphine dose can be achieved in most patients within 3 weeks, and while the optimal hourly dose varies, it nearly always falls between 3 and 7 mg/hour.

The protocols used in the TOLEDO study for starting treatment and reducing oral medication were fixed and standardized; in clinical practice, a greater variation occurs. The TOLEDO protocol, which resulted in clinically meaningful improvements in OFF periods and good tolerability, had been recommended by a panel of experts experienced with apomorphine, some of whom also participated in the questionnaire.

Apomorphine infusion therapy should be individualized for each patient—including initiation, optimal dose, reduction in oral medications, and monitoring—and should focus on the patient’s treatment goals; dyskinesias in particular should prompt attempts to reduce oral medication as much as possible but gradually, and to increase the hourly apomorphine flow rate. Good communication greatly improves the likelihood of successful outcomes and patients should be encouraged to take control of the management of their pump, including inserting the needle under the skin of the abdominal wall.

An advantage of apomorphine infusion therapy over other device-aided treatments is that it can be discontinued much more easily if results are unsatisfactory. In this regard, it is more appropriate to compare the withdrawal rates reported with apomorphine to those of oral dopamine agonists rather than to those reported from other device-aided therapies, such as gastro-jejunostomies.

A retrospective study has suggested that the best candidates for apomorphine infusion are patients with a poor health-related quality of life and marked motor fluctuations (Meira et al. 2021); predictors of discontinuation were: severe dyskinesias, a poor OFF state, poorer psychological status, shorter disease duration, and male gender. A prospective analysis of the Thai Apomorphine Registry found that predictors of discontinuation included absence of a full-time caregiver, OFF-time reduction of < 3.5 h, and Non-motor Symptoms Questionnaire scores at initiation of ≥ 9.5 points (Phokaewvarangkul et al. 2021). A long-term study over six years at a single center in Denmark found that adverse events and initial disappointment with the effect of the treatment were common early complaints but these were manageable with support and encouragement and did not lead to discontinuation (Henriksen and Staines 2021). ^32^ Extra support in the first months of treatment for patients experiencing initial difficulties with the treatment can make the difference between long-term success and failure. If OFF periods are not adequately controlled in the early stages it is also important to continue to incrementally increase the apomorphine hourly dose and reduce oral medication when dyskinesias become troublesome.

The apomorphine pump is much less expensive than enteral levodopa infusion or DBS, avoids the need for surgery and does not require a large team for long-term management. If patients need to move to residential care, apomorphine infusion can often be continued.

In summary, apomorphine infusion is an effective, well-tolerated and minimally invasive device-aided treatment for PD patients with refractory levodopa response fluctuations that cannot be managed by adjuvant oral or transdermal medication and which can often be started without the need for hospital admission. To achieve the best outcomes, patients should be referred to specialist centers as soon as motor fluctuations become difficult to manage and apomorphine considered as a treatment option. Successful treatment requires commitment from patients and their families and continuing encouragement from their doctors and nurses, particularly in the early months of therapy.

### Supplementary Information

Below is the link to the electronic supplementary material.

Supplementary file1 (DOCX 44 KB)

Supplementary file2 (DOCX 24 KB)

Supplementary file3 (DOCX 29 KB)

## Data Availability

The datasets generated during and/or analyzed during the current study are available from the corresponding author on reasonable request.
