# Supplementary material for: Practical use of apomorphine infusion in Parkinson’s disease: lessons from the TOLEDO study and clinical experience
Source: J Neural Transm (Vienna). 2023 Sep 1;130(11):1475–84. doi: 10.1007/s00702-023-02686-7 (PMC10645621; doi:10.1007/s00702-023-02686-7)
Supplement: Supplementary file 1 — Supplementary file1 (DOCX 44 KB) [file 702_2023_2686_MOESM1_ESM.docx]

##### **Apomorphine Practical Use Questionnaire**

##### **ADVISOR NAME:**

##### **COUNTRY OF PRACTICE:**

##### **Please state any local / national guidelines you follow:**

**Please provide links to these guidelines:**

### Apomorphine Practical Use Questionnaire

#### 1. Is a trial of apomorphine performed before commencing treatment?

No  Yes *If* ***yes****, please answer the questions below:*

1. How is the trial commenced?  Inpatient  Outpatient  Both
2. Is an anti-emetic usually used during the trial period?  No  Yes
3. Name of usual anti-emetic used:       Dosage:       Frequency:
4. Usual hourly apomorphine dose during trial:       mg Length of trial period:

Additional comments around the trial period:

#### 2. How long before starting apomorphine are anti-emetics usually started?

1 day  2 days  3 days

4 days  5 - 7 days  > 7 days

If this is variable, please explain the factors that determine how long before initiation that anti-emetics are started:

Name of usual anti-emetic used:       Dosage:       Frequency:      

#### 3. How soon after commencing apomorphine is domperidone / the anti-emetic usually stopped?

1 – 3 days  4 – 7 days  8 – 14 days

15 – 21 days  > 21 days  Rarely able to stop

If this is variable, please explain the factors that determine stopping the anti-emetic:

#### 4. Are patients generally commenced on apomorphine as inpatients or outpatients?

Inpatient admissions  Outpatient / day case  Both

If both, what proportion of patient’s are commenced as inpatients : outpatients?       :

Inpatients Outpatients

#### 5. What is the usual starting dose of apomorphine you commence your patients on (day 1)?

**Apomorphine naïve Those previously on**

**patients apomorphine pen**

Up to 1mg

1.01mg – 2mg

2.01mg – 3mg

3.01mg – 4mg

4.01mg – 5mg

5.01mg – 8mg

>8mg

Up to 1mg

1.01mg – 2mg

2.01mg – 3mg

3.01mg – 4mg

4.01mg – 5mg

5.01mg – 8mg

>8mg

If this is variable, please explain the factors that would determine the starting dose:

#### 6. On average, how long does it usually take to reach a stable dose?

Up to a week  > 1 week – 2 weeks  > 2 weeks – 3 weeks

> 3 weeks – 4 weeks  > 4 weeks – 5 weeks  > 5 weeks

> 6 weeks – 7 weeks  > 7 weeks – 8 weeks  > 8 weeks

If this is variable, please explain the factors that would determine the time to stable dose:

#### 7. What is the usual stable hourly dose you aim for in your patients?

1mg – 1.99mg  2mg – 2.99mg  3mg – 3.99mg

4mg – 4.99mg  5mg – 5.99mg  6mg – 6.99mg

7mg – 7.99mg  8mg – 8.99mg  9mg or more

If this is variable, please explain the factors that would determine the stable dose:

#### 8. On average, how many hours per day are patients usually administered apomorphine infusion?

Up to 6 hours  > 6 – 8 hours  > 8 – 10 hours

> 10 – 12 hours  > 12 - 14 hours  > 14 - 16 hours

> 16 – 18 hours  > 18 – 23 hours  24 hours (entire day)

If this is variable, please explain the factors that would determine the length of infusion

In what scenarios would a patient be administered apomorphine overnight?

#### 9. When down-titrating or stopping concomitant medications, what is the usual order that you would reduce/stop the concomitant medications? What is the approximate speed of this process?

**Please number each drug class in order of which would be Please given an indication of the speed of
reduced/stopped first (1) to last (5) using the drop down function this process using the drop down function**

Dopamine agonists [  ] [ ]

MAO inhibitors [ ] [ ]

COMT inhibitors [ ] [ ]

Levodopa [ ] [ ]

Other [ ] [ ]

**What factors determine the reduction of concomitant medications and the speed of this?**

#### 10. Do you use the bolus function?

No  Yes *If* ***yes****, please describe when the bolus function is used:*

What is the usual bolus dose set?      mg

How often is the bolus function utilised?

#### 11. After apomorphine has been commenced, how often are patients followed up..

**By their consultant?**  **By their nurse?**

Weekly  Weekly

Fortnightly  Fortnightly

Monthly  Monthly

Every 2 months  Every 2 months

Every 3 months  Every 3 months

Every 6 months  Every 6 months

Other:        Other:

#### 12. What parameters are monitored during treatment?

***Frequency: If frequency is “Other”:***

ECG

Coombs Test

Haemoglobin

Coombs Test

Reticulocyte count

Blood pressure

Other(s):

Other(s):

Other(s):

Other(s):
