# Supplementary material for: Practical use of apomorphine infusion in Parkinson’s disease: lessons from the TOLEDO study and clinical experience
Source: J Neural Transm (Vienna). 2023 Sep 1;130(11):1475–84. doi: 10.1007/s00702-023-02686-7 (PMC10645621; doi:10.1007/s00702-023-02686-7)
Supplement: Supplementary file 2 — Supplementary file2 (DOCX 24 KB) [file 702_2023_2686_MOESM2_ESM.docx]

**S2:** Summary of centres who responded to the questionnaire.

1. Filip Bergquist, Professor and Chief Physician Department of Pharmacology, University of Gothenburg, Sweden
2. Roongroj Bhidayasiri, Chulalongkorn Centre of Excellence for Parkinson’s Disease & Related Disorders, Department of Medicine, Faculty of Medicine, Chulalongkorn University and King Chulalongkorn Memorial Hospital, Thai Red Cross Society, and The Academy of Science, The Royal Society of Thailand, Bangkok, Thailand
3. Espen Dietrichs, Professor, Department of Neurology, University of Oslo, Oslo, Norway
4. Pedro Garcia-Ruiz, Associate Professor of Neurology, Universidad Autonoma de Madrid and Head of Movement Disorders Unit at Foundation Jimenez Diaz, Madrid, Spain
5. Tove Henriksen, Movement Disorder Clinic, University Hospital of Bielenberg, Copenhagen, Denmark
6. Regina Katzenschlager, Department of Neurology and Karl Landsteiner Institute for Neuroimmunological and Neurodegenerative Disorders, Klinik Donaustadt, Vienna, Austria
7. Andrew Lees, University College London Institute of Neurology and the National Hospital, Queen Square, London, UK
8. Simon Lewis, Consultant Neurologist and Professor of Cognitive Neuroscience, University of Sydney and Clinical Director of the Ageing Brain Clinic and Director of the Parkinson's Disease Research Clinic at the Brain and Mind Centre, Sydney, Australia
9. Patricia Pita Lobo, Instituto de Medicina Molecular, Faculdade de Medicina, Universidade de Lisbon, and Departamento de Neurociências e Saúde Mental, CHULN, Lisbon, Portugal.
10. Friederike Sixel-Döring, Paracelsus-Elena-Klinik, Kassel and Philipps University of Marburg, Marburg, Germany.
11. Malcolm Steiger, Consultant Neurologist and Director of Research and Development, The Walton Centre for Neurology, Liverpool UK
12. Fabrizio Stocchi, Professor of Neurology, Director of the Parkinson’s Disease and Movement Disorders Research Centre, and Director of the Drug Development Research Centre at the University and Institute for Research and Medical Care IRCCS San Raffaele Pisana, Rome, Italy
13. Marc Vérin, Head of Institute of Clinical Neurosciences, University Hospital Pontchaillou, Rennes, France
