# Supplementary material for: Practical use of apomorphine infusion in Parkinson’s disease: lessons from the TOLEDO study and clinical experience
Source: J Neural Transm (Vienna). 2023 Sep 1;130(11):1475–84. doi: 10.1007/s00702-023-02686-7 (PMC10645621; doi:10.1007/s00702-023-02686-7)
Supplement: Supplementary file 3 — Supplementary file3 (DOCX 29 KB) [file 702_2023_2686_MOESM3_ESM.docx]

**S3: Quick reference summary of key practical recommendations**

| 1. **Setting for apomorphine infusion initiation** | Outpatient initiation is straightforward but there may be specific reasons why hospital admission may be preferable. Inpatient initiation may make the patient feel more confident, allow repeated training in handling the device, and give staff the opportunity to make early observations on tolerability or potential difficulties. Initiation can usually be completed over 5–10 days, after which less frequent visits are required to allow for further adjustments. |
| --- | --- |
| 1. **Anti-emetic use when initiating apomorphine** | Clinicians should review the patient's medical history, medications, and ECG. If there is no prior history of nausea, domperidone may not be needed. It is also contraindicated in severe hepatic impairment, in conditions where cardiac conduction is or could be impaired, and when co-administered with QTc-prolonging medications or potent CYP3A4 inhibitors.^1^ If needed (and available), domperidone should be used at the lowest effective dose for the shortest possible time, as per EMA recommendations. |
| 1. **Titration regimen during the first 10 days** | There are various options for titration in clinical practice: a quick titration, most commonly as an inpatient, starting at 0.5–1.0 mg/hour, with increments of 0.5–1.0 mg every few hours , depending on tolerability or increased dopaminergic effects such as dyskinesias; or a slower titration, more suited for outpatient initiation, with increments of 1.0 mg/hour at intervals that depend on individual response and tolerability but usually take one week. If no anti-emetic is available the incremental increase may need to be slower. |
| 1. **Time to stable dose and stable dose achieved** | Each patient’s stable apomorphine dose will depend on individual efficacy and tolerability, and on their wishes In contrast to the randomized study, in clinical practice, there is no time limit to dose adjustments and this may take longer than the 4 weeks specified in the study protocol. The aim is an effective, well tolerated dose with individually adjusted concomitant oral medication. |
| 1. **Reductions and discontinuation of concomitant anti-PD medication** | If dopaminergic side effects, such as increased dyskinesias, occur after starting apomorphine then concurrent oral medication should be gradually reduced, usually in the order suggested in Table 1. Sudden withdrawal of any oral anti-Parkinsonian medication is not recommended, and in the case of dopamine agonists may precipitate dopamine agonist withdrawal syndrome.  Dyskinesia severity determines the optimal degree of dose reduction. To treat OFF periods, the reduction in oral drugs may only need to be small, however in a patient with troublesome dyskinesia, gradual withdrawal of all daytime oral medication may be required with the exception of a night-time and early morning levodopa dose.  If the patient continues to experience tolerability issues despite oral medication reduction, the apomorphine dose should be decreased. Conversely, if side effects are reduced following oral drug alterations, but the clinical effect of apomorphine infusion is still sub-optimal, apomorphine should be slowly increased.  In patients with troublesome nocturnal OFF symptoms, 24-hour use may be helpful. If apomorphine infusion is only used during daytime, nocturnal parkinsonian symptoms may be alleviated by using levodopa at bedtime  If dyskinesias are still troublesome while using apomorphine infusion, amantadine can be introduced or increased.  During the early treatment phase, regular clinic visits are needed to achieve the best long-term results. |
| 1. **Hours of infusion** | The primary aim is for a waking day infusion of around 16 hours, depending on each patient’s personal schedule and preference. In patients with troublesome nocturnal OFF symptoms, 24-hour use may helpful. |
| 1. **Monitoring of patients established on treatment and management of adverse events** | One advantage of apomorphine infusion over other device-aided therapies, such as enteral levodopa infusion, is that it is easy to discontinue if the response is unsatisfactory or if intolerable adverse events occur.  Skin nodules and irritation are relatively straightforward to manage and are rarely a cause for discontinuation.  Orthostatic hypotension (OH) occurs in ≤1% of patients and is usually mild and transient. Monitoring patients´ fluid intake, particularly in hot climates, may be helpful.  Haemolytic anaemia, indicated by a falling haemoglobin level, a positive Coombs test and other characteristic laboratory changes (such as increased lactate dehydrogenase and decreased haptoglobin), is a rare serious adverse event associated with apomorphine infusion. In clinical practice, in addition to blood checks, clinical signs should be monitored and patients should be educated about symptoms of anaemia. |
| **Bolus function** | Occasional use of the bolus function of the apomorphine infusion pump is recommended, if necessary, since it increases the flexibility of treatment and empowers the patient (or carer if they administer this). Patients should be educated on situations where this may be appropriate. Frequent use (>3 times daily) suggests the hourly flow rate should be increased |

1. European Medicine Agency (2014) PRAC recommends restricting use of domperidone 2014. Available from: <http://www.ema.europa.eu/docs/en_GB/document_library/Referrals_document/Domperidone_31/Recommendation_provided_by_Pharmacovigilance_Risk_Assessment_Committee/WC500162559.pdf>
